# Geographies of Care: The Catholic Church in Poland’s Assistance to Refugees from Ukraine During Russia’s Invasion of Ukraine

**DOI:** 10.1007/s10943-022-01729-9

**Published:** 2022-12-31

**Authors:** Franciszek Mróz

**Affiliations:** Department of Tourism and Regional Studies, Institute of Geography, Pedagogical University of Krakow, ul. Podchorążych 2 (room 526), 30-084 Kraków, Poland

**Keywords:** Catholic Church in Poland, Care, Geographies of care, Refugees from Ukraine, Religion, Russian invasion of Ukraine

## Abstract

The purpose of the article was to analyze the collected empirical material in the form of in-depth interviews, observations, statistical data, and numerous accounts of the assistance of the Catholic Church in Poland in the first 8 months of Russia’s invasion of Ukraine. The results of the survey revealed that the Catholic Church’s activities and support to Ukrainians were carried out on many levels: charitable—mainly material, financial and social housing assistance, psychological, educational, and medical. All Catholic parishes and almost all women’s and men’s convents and monasteries in Poland, Caritas Poland, as well as dozens of church institutions, joined in helping refugees from Ukraine.

## Introduction

The turn of the second and third decades of the twenty-first century saw upheavals in the lives of almost everyone on earth. The global upheaval began a few weeks after the discovery of the SARS-CoV-2 in Wuhan, China, in late 2019. As a result of the COVID-19 pandemic, more than 6.57 million deaths reported to the WHO had been registered by November 1, 2022, while the number of infected had already exceeded more than 627.57 million (World Health Organization, 2022, November 1). The dynamics of change in the geographic space of the world—especially Europe—has also taken a drastic turn in recent months, following Russia's invasion of Ukraine, which began on February 24, 2022. The war in Ukraine has triggered a tremendous amount of geopolitical, social, and economic changes that occurred both on the global scale and on the homestead. These changes have not been limited to Europe.

The text presented here is part of the research related to religion and health, particularly geographies of care (Conradson, 2003; England, 2010; Iacovone et al., 2020; McEwan & Goodman, 2010; Milligan & Power, 2009; Moosa-Mith, 2016; Mróz & Roszak, 2022; Springer, 2020). The social, economic, religious, and geopolitical changes that have taken place in the world in the last 3 years have prompted a number of studies related to care, which has become a very pressing issue on many spatial scales (Lawson, 2008).

It is also another attempt to confirm the legitimacy of undertaking human geography research related to the geography of care, the geography of brotherhood (Jakubowski, 1985; Plit, 2005), and the geography of neighborly love (Morrison et al., 2013)—in response, as it were, to much work related to the geography of war, or the geography of crime (Georges, 1978; Lawson, 2008). An important impetus for the research was personal contact with dozens of priests, monks and nuns, and Catholic volunteers showing assistance to Ukrainians who have been forced to leave their homes since Russia invaded their homeland.

The research problem solved in the paper has not yet been addressed in the literature. However, attention should be paid to the recent studies conducted by Oviedo et al., (2022) and Roszak, (2022), who examined the traumatic experiences of war refugees from Ukraine and identified their coping strategies and the resilience they achieved through their religiosity, as well as on Bouchard's research and on the psychotraumatology of war in Ukraine of others (Bouchard et al., 2022).

The study presents the results of research focused on the assistance of clergy, religious congregations, volunteer-based organizations, and many institutions of the Catholic Church in Poland to the large group of refugees from Ukraine. The analysis was conducted with reference to 8 months of 2022—from the moment of Russia's invasion of Ukraine (from February 24, 2022 to the end of October 2022). It should be noted, however, that already before the outbreak of war in Ukraine in February 2022, many Catholic parishes in Poland and Caritas Poland began preparing aid—mainly material assistance and places for possible refugees from Ukraine (Jałowiczor, 2022). According to forecasts by the Polish Ministry of Internal Affairs and Administration, even before the outbreak of war—in the wake of the Russian Federation's invasion of Ukraine—up to 1 million people could seek refuge in Poland. From the data provided by the Border Guard in Poland, we know that from the beginning of the Russian–Ukrainian war until October 31, 2022, more than 7.4 million refugees from Ukraine crossed the Polish–Ukrainian border (Straż Graniczna, 2022, October 31; Serwis Rzeczypospolitej Polskiej; 2022, October 20).

The aim of the research was to answer the following research question: what were the forms and volume of assistance provided by the Catholic Church in Poland to war refugees from Ukraine arriving in Poland and humanitarian aid transferred to Ukraine? The presented work can make an important contribution to further research related to the geography of care in relation to the war-affected residents of Ukraine.

## Materials and Methods

Learning about the role of the Catholic Church in Poland to refugees from Ukraine during Russia's invasion of Ukraine required intimate research, as well as interviews and observations. A total of 26 in-depth interviews were conducted with priests, seminarians, employees of diocesan branches of Caritas, monks and nuns, and volunteers helping Ukrainian refugees in Poland. Valuable data were obtained from the Witold Zdaniewicz Institute for Statistics of the Catholic Church SAC, which in April 2022 published the report “Assistance to refugees from Ukraine by Catholic parishes and religious orders in Poland. Report for the period from February 24 to March 31, 2022” (Organek, Sadłoń,^2022^). The report is based on research conducted from March 23 to April 2, 2022, and involving 1,338 Polish Catholic and Greek Catholic parishes (12.9% of all parishes in Poland), as well as 110 female convents (out of 155 of all those operating in Poland) and 224 male monasteries (25% of all religious houses operating in Poland) (Organek, Sadłoń, 2022).

Data and information were also independently obtained from the Central Statistical Office, the Secretariat of the Conference of Major Superiors of Female Religious Congregations, the Secretariat of the Conference of Major Superiors of Male Religious Congregations, the curia of Catholic dioceses in Poland, Caritas Poland, and diocesan branches of Caritas. A review of Catholic dailies and weeklies (Catholic weekly “Gość Niedzielny,” Catholic weekly “Niedziela,” and Catholic daily “Nasz Dziennik”) also provided much information. A detailed review was also conducted of information posted on the websites of diocesan branches of Caritas Poland, higher seminaries, almost all religious congregations in Poland, and on the social media platforms (Facebook, Twitter and Instagram) of religious orders—a total of 59 Web sites of male religious congregations and 124 websites of female religious congregations, as well as over 200 Catholic parishes in Poland. Data on the number of refugees from Ukraine in Poland were obtained from the Border Guard Headquarters, Operational Duty Service Department, Border Management, and data from the United Nations High Commissioner for Refugees.

## Results

**Fig. 1:**
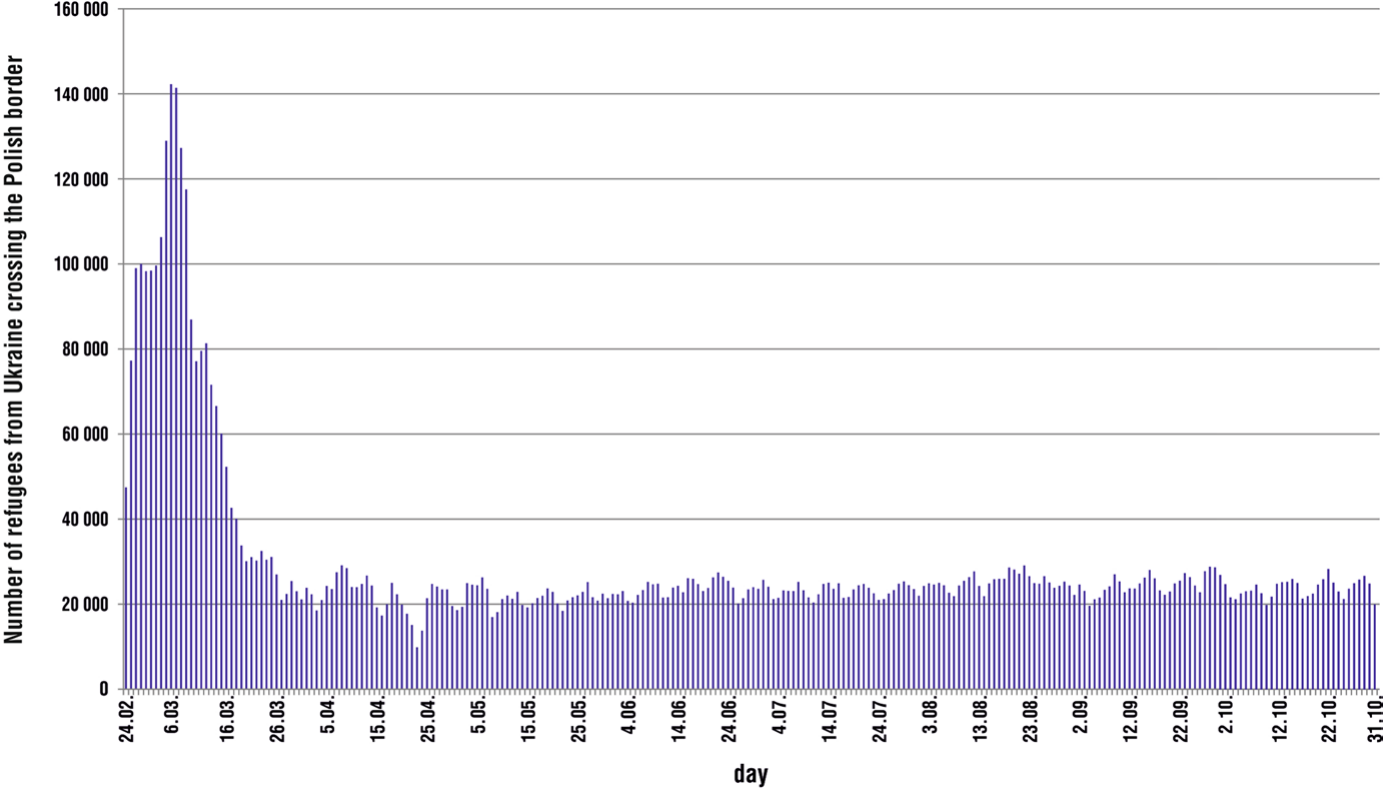
Refugees from Ukraine crossing the Polish–Ukrainian border from 24.02.2022 to 31.10.2022 by day. *Source*: own elaboration based on data from the Border Guard Headquarters in Poland

Poland is the country that has played the most important role in receiving refugees from Ukraine since the beginning of the Russian aggression against Ukraine. According to the Border Guard in Poland, the number of refugees arriving in Poland from Ukraine between February 24 and October 31, 2022 was 7.429 million—mostly women and children (according to the UNHRC, 95% of refugees were women; Operational Data Portal by UNHCR, 2022, October 31; Straż Graniczna, 2022, October 31). The number of refugees crossing the Ukrainian–Polish border at eight border crossings has increased sharply since the second week of the war. The record daily number of refugees was registered on March 6—142,298 people and March 7—141,415 people (Serwis Rzeczypospolitej Polskiej; 2022, October 20 (Fig. 1). During the first month of the war (from 24 February to 23 March), 2,198,749 refugees from Ukraine arrived in Poland—increasing Poland’s population by 5.8% (at the end of 2021, Poland's population was 37.908 million) (Serwis Rzeczypospolitej Polskiej; 2022, October 20) (Fig. 1).

It should be noted that by the decision of the Polish Government, refugees from Ukraine were allowed to cross the Ukrainian–Polish border even without identity documents, and were exempted from quarantine due to the COVID-19 epidemic. After the first month following the outbreak of war, the number of refugees from Ukraine crossing the Ukrainian–Polish border decreased, with an average of more than 25,000 people crossing the Ukrainian–Polish border every day for the next 7 months (Fig. 1).

**Fig. 2:**
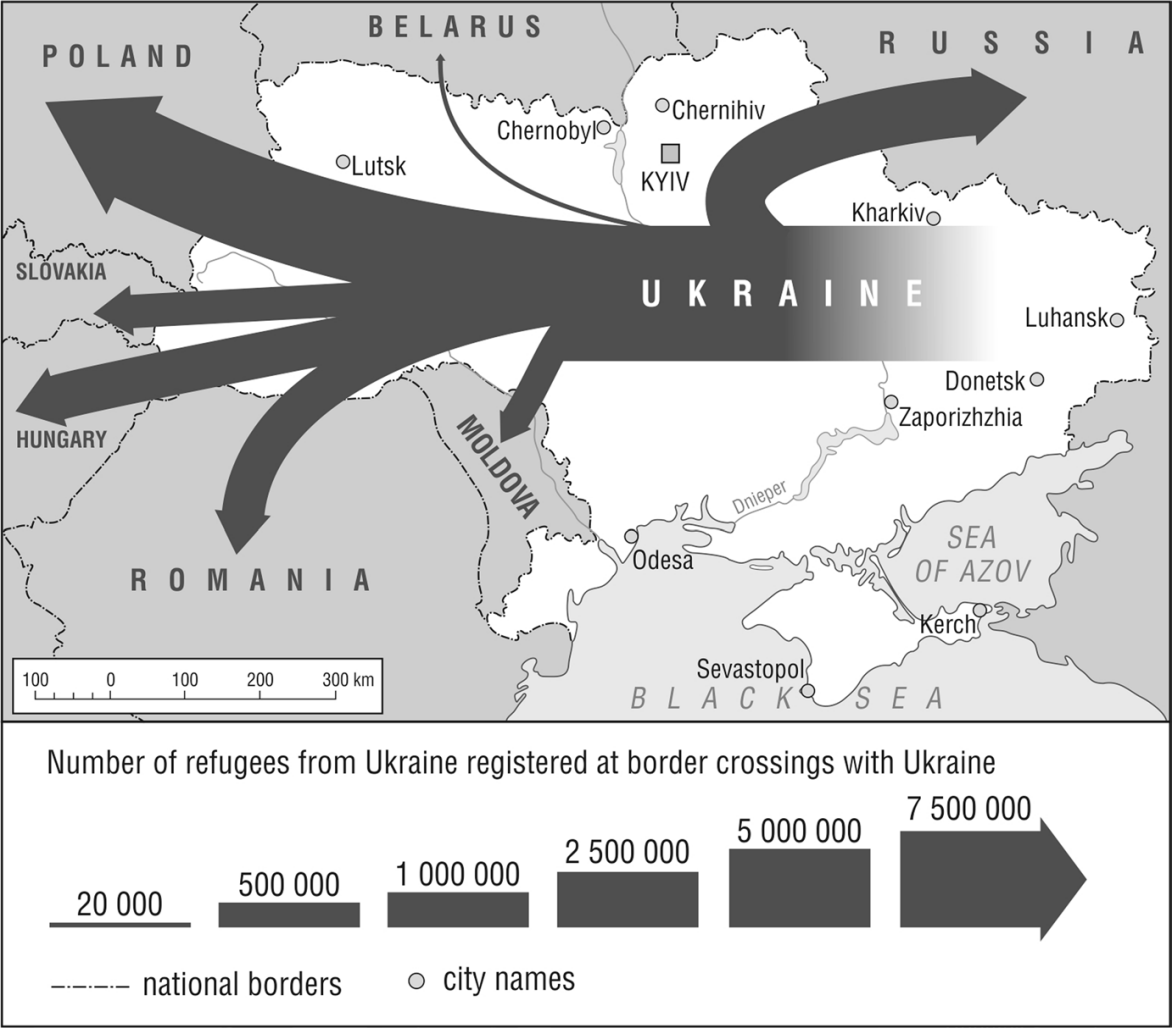
Inflow of refugees from Ukraine to neighboring countries (24.02.-1.11.2022). *Source*: author’s elaboration based on data from UNHCR: https://data.unhcr.org/en/situations/ukraine (accessed 1.11.2022)

Such a huge influx of refugees as to Poland was not encountered after World War II by any country in Europe—for comparison, from 24 February to 1 November 2022, Hungary received 1,670,028, Romania 1,460,854, Slovakia 900,724, and Moldova 678,326 refugees from Ukraine (Operational Data Portal by UNHCR, 2022, November 1) (Fig. 2).

The war in Ukraine has also resulted in one of the fastest displacements of children since the Second World War. According to UNICEF, in the first month of the war in Ukraine alone, 4.3 million children (more than half of the country's estimated 7.5 million child population) were displaced—of which more than 1.8 million children came to neighboring countries as refugees, and 2.5 million resettled with their parents within Ukrainian territory; UNICEF, 2022, March, 24).

On the second day after Russia's invasion of Ukraine, the Permanent Council of the Polish Bishops' Conference held an emergency online meeting on February 25, 2022. The Council issued a communiqué in which the Polish bishops assured prayers for “the millions of people affected by the brutal military attack by Russia,” condemned in the strongest terms “the barbaric decision of the Russian president to initiate military action against Ukraine,” called “for repentance and an end to military actions that claim many lives, including civilians,” and appealed to the faithful in Poland to “open to sisters and brothers from Ukraine […] homes, hostels, diocesan, parish and retreat houses and any place where help can be provided to people in need” (Rada Konferencji Episkopatu Polski ds. Migracji, Turystyki i Pielgrzymek, 2022).

The influx of refugees from Ukraine to Poland caused a huge mobilization of Polish society and confirmed the durability and scale of the Polish tradition of solidarity, hospitality, and religiosity (Oviedo et al., 2022). Just a dozen hours after the Russian invasion of Ukraine, the first volunteers appeared at eight road border crossings (Dorohusk–Jagodzin, Zosin–Uściług, Dołhobyczów–Uhrynów, Hrebenne–Rawa Ruska, Budomierz–Hrushev, Korczowa–Krakowiec, Medyka–Szeginie and Krościenko–Smolnica; Straż Graniczna, 2022, October 31), and at the Przemyśl railroad station (Przemyśl–Mościska railroad border crossing) ready to help Ukrainians fleeing their homeland from the effects of war. During the following days, “tents of hope” were set up at border crossings by Caritas and a great many organizations (including Catholic ones) bringing aid and respite to refugees crossing the border after waiting for hours. In these places, they received hot meals and the most urgent personal hygiene products, and were then redirected to reception points or taken by volunteers to assured accommodations in Poland.

The involvement of the Polish public—volunteers, uniformed services, public and non-public entities, and the Catholic Church (clergy, religious, church institutions) grew with each passing day of the war in Ukraine. Helping refugees proved crucial, especially in the first weeks of the refugee wave, when there was a lack of reception points and, above all, coordination. The reception of Ukrainians in thousands of Polish homes, shelters, accommodation facilities, fire stations, sports halls, monasteries of male and female religious orders, Caritas centers, pilgrim's homes, seminaries, catechism classrooms, parish houses, parsonages, centers belonging to Catholic movements and communities, bishops' palaces, and other specially prepared facilities with sanitary and heating facilities. This was possible thanks to the gigantic effort and solidarity of millions of Poles, government and local authorities, NGOs, and the Catholic Church. It should be noted that no large refugee camps were established in Poland.

Subsequent weeks of war in Ukraine brought new solutions and forms of aid shown to refugees in Poland and humanitarian aid transferred to Ukraine.

A detailed analysis of the material, financial, medical, educational, psychological and spiritual assistance shown by the clergy, consecrated persons, the faithful and many entities of the Catholic Church in Poland to refugees from Ukraine has proved very difficult due to the multiplicity of such activities. This makes it all the more enormously complex to accurately the amount of aid expressed in monetary amounts and the number of beneficiaries. However, the author of the presented study found it necessary to present the results of the research as synthetically as possible, summarizing the assistance of the Catholic Church in Poland in the first eight months of the war in Ukraine.

Bishop Krzysztof Zadarko—chairman of the Council of the Polish Bishops' Conference on Migration, Tourism and Pilgrimages during an interview with the Author of the presented work—stressed that in the first weeks of the war in Ukraine, “there was no parish in Poland that did not provide assistance to refugees.” It is worth noting that at the beginning of 2022, there were 10,448 Catholic parishes in Poland (Organek, Sadłoń, 2022), while there were a total of about 30,300 consecrated persons—16,307 nuns in female active congregations, about 1,250 nuns in contemplative monasteries, and 11,020 religious (the number of male religious houses is 895 and the number of female religious houses is 1,985 (Konferencja Wyższych Przełożonych Żeńskich Zgromadzeń Zakonnych, 2022a; Serwis Informacyjny Konferencji Wyższych Przełożonych Zakonów Męskich w Polsce).

### Material, Financial and Charitable Assistance

The main and most common forms of assistance provided to refugees from Ukraine were financial aid, material assistance, and the provision of accommodation. Among a group of 1113 Catholic parishes surveyed by the Institute of Catholic Church Statistics, 94.4% of the parishes surveyed indicated that they had provided financial resources to refugees from Ukraine (the average value of assistance provided by 1 parish was PLN 9871), and 73.9% of the parishes surveyed provided material gifts (the average value of assistance provided by 1 parish was PLN 10,213) (Organek, Sadłoń, 2022, 7).

On Sunday, February 27, 2002, and Ash Wednesday, a financial collection was held in all Catholic parishes in Poland after each Mass. In the first 3 weeks of the war in Ukraine alone (up to 13 April 2022), Catholic and Greek Catholic parishes in Poland collected and transferred financial and material aid worth at least PLN 209 million to Ukrainian refugees residing on Polish territory, male religious orders collected PLN 29 million and female religious orders PLN 4.3 million (Katolicka Agencja Informacyjna, 2022, April 13).

The vast majority of the more than 830 (Sadłoń, 2015) charitable institutions of the Catholic Church in Poland joined in helping refugees fleeing the effects of bloody attacks by Russian troops. The largest scope of assistance was (and still is) carried out by Caritas Poland in cooperation with 41 diocesan Caritas branches of the Roman Catholic Church (a total of 32 Centers for Migrants and Refugees), Caritas of three Greek Catholic dioceses, parish Caritas branches, the Polish section of the Pontifical Association to Aid the Church in Need, the Union of Polish Maltese Cavaliers and the Maltese Medical Service, and the Knights of Columbus. Caritas Poland constantly conducts financial and in-kind collections, including the “Parcel for Ukraine” campaign. It is thanks to these collections that Caritas has provided refugees from Ukraine with in-kind gifts with a total estimated value of more than PLN 140 million, (Caritas, 2022; October 31) and more than 40,000 parcels with a total value of more than PLN 14 million have been prepared for those most in need.

Diocesan Caritas shares took care of the relocation of refugees, set up accommodation and volunteer bases, and set up aid stations—“tents of hope”—at railroad stations in major cities and at border crossings, where volunteers were on duty day and night, and provided additional assistance. In total, about 20,000 volunteers joined in helping refugees organized by Caritas Poland, of whom about 1,300 helped at the Polish–Ukrainian border. The 210 diocesan Caritas branches in Poland provided assistance to refugees (Caritas, 2022; October 31). The fruit of Caritas Poland's cooperation with partners affiliated with the international federation Caritas Internationalis, primarily Caritas in Ukraine—Caritas-Spes and Caritas Ukraine, as well as the American Catholic Relief Services, Caritas Switzerland and Caritas Deutschland, was the establishment of two logistics centers on the premises of Caritas Archdiocese of Przemyśl and Lublin, as well as a cross-border hub of more than 3,000 sq. m. with a warehouse managed by Caritas Archdiocese of Lublin staff. This significantly improved the speed and fluidity of humanitarian transports to Ukraine. Each of the diocesan branches of Caritas in Poland additionally conducted other campaigns to help refugees from Ukraine.

Particularly noteworthy in this regard are the initiatives and assistance carried out (and still) undertaken by diocesan branches of Caritas—Caritas of the Lublin Archdiocese, Caritas of the Przemyśl Archdiocese and Caritas of the Zamość-Lubaczów Diocese, due to the fact that their spatial scope of influence covers the Polish–Ukrainian borderland and there are road and rail border crossings with Ukraine in these dioceses. The largest number of refugees crossed the border at the Medyka–Szeginie border crossing and arrived by train from Lviv and Kiev to Przemyśl (at the Border Guard Point in Medyka, the total number cleared by 31.10.2022 was 2,217,171) (Serwis Rzeczypospolitej Polskiej; 2022, October 31). The volume and scale of assistance to Ukrainians of the Lublin, Przemyśl and Zamość-Lubaczów branches of Caritas from the beginning of the war until the end of October 2022 was gigantic. It was (and still is) aid to refugees arriving in Poland, as well as humanitarian aid transferred to Ukraine. During the period under review, Caritas of the Archdiocese of Przemyśl organized 834 trucks and 334 buses with humanitarian aid transferred to Ukraine—mainly to the residents of Lviv, Ternopil, Kharkiv, Chernihiv, Dnipro, Kamieniec Podolski, Kiev, Odessa, Vinnitsa, Zaporizhzhya, Zhytomyr, and to refugees in western Ukraine. There were about 27,000 pallets of products with food, school supplies, hygiene, medical and first aid items, hospital and field beds, mattresses, sleeping pad, sleeping bags, blankets, children's backpacks, fire extinguishers, strollers and cribs, flashlights and water tanks for shelters. The weight of donated support—food and hygiene products amounted to 1,311,000 kg. As part of the “Parcel for Ukraine” program, 73,000 packages were transferred to Ukraine. Thanks to Przemyśl Caritas, 280 generators and 4 ambulances were transferred from Przemyśl to Ukraine. In addition, Przemyśl Caritas, in cooperation with 150 volunteers and seminarians from the Przemyśl Theological Seminary, distributed more than 40,000 sandwiches to refugees daily. It also donated 45 wheelchairs for migrants with disabilities, provided round-the-clock care and treatment to 25 chronically ill people from Ukraine, and sheltered 20 mothers with children at the Mother and Child Home (Caritas Archidiecezji Przemyskiej, 2022, October 31). In turn, Caritas of the Lublin Archdiocese organized 231 trucks and 156 buses of humanitarian aid donated to Ukraine with a total value of about PLN 23,940,000 (Caritas Archidiecezji Lubelskiej, 2022, October 31). Thanks to the Lublin branch of Caritas, 24,798 refugees benefited from emergency assistance at the House of Friendship in Lublin, and nearly 500 people benefited from 24-h assistance at Caritas centers (Caritas of the Lublin Archdiocese, 2022, October 31).

The aid and support provided to Ukrainian citizens by Caritas of the Zamość-Lubaczów Diocese were also notable. From data received from Fr. Marcin Jakubiak—Director of Caritas of the Zamość-Lubaczów Diocese, we know that from the day of the outbreak of war in Ukraine until October 31, 2022, the estimated volume of financial support to help Ukraine from external funds that Caritas of this diocese acquired amounted to PLN 8,573,916.06, while systematic humanitarian convoys with aid directed to the population remaining in Ukraine (Lviv, Zhovkva, Kharkiv, Kiev)—a total of more than 550 buses and trucks with humanitarian aid with a total value of about PLN 40 million.

It is worth noting that Caritas Poland's commitment to Ukrainian refugees was honored with the Res Publica award on October 29, 2022, in Mondovì, Italy. Religious congregations in Poland have not remained indifferent to the refugees fleeing Ukraine. Female religious congregations have joined enormously in providing assistance related to housing (91.8% of female congregations have taken in refugees for more than 1 week), as well as providing material resources (90% of congregations) and financial aid (85.5% of congregations).

According to data received from the Secretariat of the Conference of Major Superiors of Female Religious Congregations, 924 female religious houses in Poland and 98 in Ukraine provided material, spiritual, psychological and medical assistance. Accommodations were organized in 498 religious houses in Poland and 76 houses in Ukraine (Konferencja Wyższych Przełożonych Żeńskich Zgromadzeń Zakonnych, 2022b). As of March 14, 2002, 3060 children, 2420 families, and more than 2950 adults had received shelter in female convents in Poland.

In contrast, the assistance of male religious congregations focused on financial aid (76.3% of the religious houses surveyed), assistance related to the provision of accommodations (64.7%), and material aid (62.3%) (Organek, Sadłoń, 2022, 10–11). As of March 17 of this year, which was the fourth week of the Russian–Ukrainian war, 156 men’s religious houses sheltered 3630 refugees from Ukraine, including 1483 children, while 4 monasteries received 61 people with disabilities, including 37 children (Katolicka Agencja Informacyjna, 2022, April 13). At the time, monks and nuns provided Ukrainians with automatic washing machines, stoves, microwave ovens, and other household appliances.

Some of the Ukrainian refugees took advantage of the accommodation opportunity for a few days, and then went on their way. But many families, especially mothers with children, are still (i.e., on October 31, 2022—the day of completion of the presented text) living in monasteries (e.g., in Kraków, Warsaw, Wroclaw, Jamna). A touching moment for the common Dominican monastery in Warsaw-Sluzew was the birth of a child whose family took refuge in this monastery. The boy was named Dominic, in honor of the founder of the Order and the patron saint of this parish.

Many monks and nuns assisted Ukrainian refugees at border crossings between Poland and Ukraine, at Caritas Tents of Hope and at train stations in Przemyśl and major Polish cities. Fr. Maciej Ziębiec CSsR—a Redemptorist, missionary, and retreatant who has been involved in a number of outreach works for many years, is currently initiating a number of activities to help refugees from Ukraine: "Mercy is the motto of my life. I have received the immensity of mercy from Christ, and I cannot imagine not living by this mercy. St. Clement Maria Hofbauer often spoke of reading the signs of the times. And for me, this reading of the sign of the times towards the poor and abandoned is precisely addressing the war. As soon as I heard about the refugees, a desire to go to the border was instantly born in me. After getting permission from my supervisor, I immediately went to the border crossing in Zosin. I arrived there on March 11 and stayed there - with short breaks – for more than two months. This time has become a part of my personal history, and I have been proud to bear its hardships. For me, a powerful experience was speaking with the refugees who came to us without really knowing what would happen to them, without knowing how they should live on. When one meets a person in whom one sees life, but that life – in the eyes, in the face, in the children they carry in their arms – is filled with suffering and thousands of questions "why us? what did we do?" – is immensely difficult. Most of the help we gave was simply to receive with great respect and peace, to give them what we have at the moment – accommodation, food, care. What was hugely important was to be with them, to give them that security just 50 meters from their war-torn country, it was important to talk to them – so that they didn't feel like people passing by. We strived to make them feel safe and cared for. Humanitarian aid transports to Ukraine also began at the border. After the closure of the tent of hope and the return to Warsaw, funds appeared from a huge number of people from different parts of the world to organize humanitarian transports to Ukraine. We organize transports with very specific aid to specific people - consulting their contents with the people in Ukraine – and so, for example, we have sent 10 ambulances, and have now purchased Honker and Jeep all-terrain vehicles, because in the hard-to-reach – muddy, swampy areas of Ukraine there is a great need for such vehicles to reach the needy with aid." O. Maciej Ziębiec CSsR (excerpted from statements made during an interview on November 13, 2022).

The monks' and nuns' knowledge of the Ukrainian language proved to be very valuable. Some of the Ukrainian refugees crossing the Ukrainian–Polish border went to specific religious houses or relief facilities run by the Catholic Church, as such contacts were given to them by monks and nuns working in Ukraine. Sister Jonatana Mach—a Seraphic Sister—from the first hours of the war in Ukraine helped refugees reaching the train station in Przemyśl:"That first week of the war was very difficult. The refugees who came to us had serious communication problems, because there was a shortage of Ukrainian speakers among the volunteers. Since I know Ukrainian, as I worked in Ukraine for many years, I was more than happy to help them with interpersonal communication. This image of people fleeing Ukraine was very tragic to me. A crowd of people. A river of people. I couldn't understand that in today's world such a situation could occur, that people would flee their homeland in fear of death. As I saw these people – mostly women with children, hungry, unwashed, immensely lost, sitting in that train station with eyes full of hopelessness – it was terrifying. When I spoke to them in Ukrainian they were very happy and grateful. They were extremely happy to ask and get the information they needed. This information was very important to them - because they asked "where can they go now?" "what can they do next?". I also tried to help these people mentally and spiritually – by talking, hugging them, and praying with them. A very poignant moment for me was the transportation of disabled people from under Kiev. These people were brought to the dormitory of the Special School and Educational Center in Przemyśl, where I work. They traveled to Poland for several days. It was remarkable to me that despite these dramatic circumstances and their disabilities, they tried to seek hope and help in a better world. Today I continue to help translate and teach Ukrainian children in school." Sister Jonatana Mach—a Seraphic Sister (excerpted from a statement during an interview on November 10, 2022).

The Union of Polish Knights of Malta and the Malta Medical Service, which operates within its structures, have also joined in helping the affected refugees from Ukraine. They organize medical and material support implemented in Ukraine and war refugees from Ukraine in Poland. The Maltese have organized aid and reception points at border crossings in Hrebenne, Krościenko and Korczowa, a relocation point for people and redistribution of donations in Kombornia, two medical aid points at railway stations in Kraków and Katowice, and 13 collection points in major Polish cities—special Maltese refugee aid centers. Some 1,300 Maltese volunteers were involved in these actions during the first 3 months of the war. The estimated value of the aid provided by the Union of the Polish Knights of Malta and the Malta Medical Service during the 7 months of the war is about PLN 66.75 million (Związek Polskich Kawalerów Maltańskich, 2022).

On April 24, refugees from eastern rite Ukraine (believers of the Orthodox Church and the Byzantine-Ukrainian Rite Catholic Church) celebrated Easter. For them, diocesan branches of Caritas with the support of Polish Hotel Holding, as well as Catholic parishes organized Easter breakfasts keeping in mind the Ukrainian character of the dishes—so the Easter tables did not lack Ukrainian borscht, festive pascha, yeast babka with nuts (called paska in Ukraine) and eggs in various forms. In turn, the Brother Albert Foundation of Radwanowice, which runs houses and occupational therapy workshops for people with intellectual disabilities, prepared places for women with children in Kraków, Radwanowice and Zawoja, as well as other towns where there are Foundation facilities.

Religious sisters and nuns also prepared warm meals, food parcels, hygiene products and medicine for Ukrainian refugees. Many religious congregations in Poland, independently of the actions taken by Caritas Poland, organized humanitarian aid transports to monasteries in Ukraine, carrying medical supplies, cleaning products, food to the areas attacked by Russian troops, power generators, sleeping bags, sleeping pads, mattresses, blankets, quilts, field beds, backpacks, clothing, underwear, power banks, flashlights, toiletries, and even bread-baking equipment—a professional baking mixer—a gift from the Oblate parish in Opole to the homeless in Kiev. In the first month of the war alone, at least 34 cars left from men's religious houses with humanitarian aid to Ukraine, carrying nearly 100 tons of donations (www.zyciezakonne.pl).

Diocesan clergy, consecrated persons, Caritas, religious fraternities, the Order of the Knights of Malta, the Knights of Columbus and volunteers from many Catholic associations joined in assisting refugees at reception points and at border crossings and train stations—especially the Przemyśl train station and in Poland's largest cities. They coordinated emergency and long-term assistance, donated in-kind aid and food, organized assistance and care for children, arranged translators, coordinated the search for accommodation and jobs, helped deal with official matters and obtain legal assistance, organized waiting rooms for mothers with children with a playroom and activities for children, assisted those in need during hospital visits, helped with transportation to places of accommodation and also provided care for animals. In dozens of parishes in Poland, parish consultation points were set up, and in the largest religious houses and shrines, such as those at Jasna Góra and the Divine Mercy Shrine in Kraków-Łagiewniki, logistics centers were established to which transports of humanitarian aid organized at other facilities abroad and in Poland arrived. For several months, several Polish parishes had “Shelves of Goodness”—stores organized in parish halls in which the only means of payment was the word “thank you,” and in which refugees from Ukraine could pick up food products, clothing, footwear, hygiene products and cosmetics free of charge. Thanks to Caritas Poland alone, 8,000 Ukrainian children participated in holiday trips and received school kits. In turn, the Knights of Columbus in Poland organized humanitarian transports to Ukrainians in need—a total of more than 40,000 food and chemical parcels (by the end of 2022, they plan to prepare and deliver a total of 100,000 gift parcels to Ukraine). Members and friends of the Knights of Columbus have donated nearly $20 million to the Solidarity Fund for Ukraine (Katolicka Agencja Informacyjna, 2022, October, 24).

Refugees from Ukraine have also found refuge in pilgrim's homes in Poland's largest shrines—including Jasna Góra, the Divine Mercy Shrine in Kraków-Łagiewniki, the Shrine of St. John Paul II in Kraków, Mount Saint Anne, Licheń, Kalisz, Kalwaria Zebrzydowska, Kalwaria Pacławska, Krasnobród, Ostrowąs, Pakość, Gdańsk Matemblewo, Rudy, Sejny, Szczepanów, Wadowice, Tuchów, and Wejherowo. Material assistance, psychological, informational and legal support, as well as play areas for children were also organized for refugees in these places.

It should be noted that many monasteries in Poland also welcomed volunteers from abroad who came to Poland to help Ukrainian refugees at border crossings or participated in humanitarian transports to Ukraine. The clergy, religious, the faithful and Catholic associations in Poland published information on social media about charity events, places to collect donations for Ukrainians, the refugee procedure, material and financial aid. On the websites of Caritas, religious orders (including the Information Service of the Conference of Major Superiors of Male Religious in Poland) there are permanent tabs “Aid for Ukraine” (“Religious for Ukraine”), where constantly updated information on aid for Ukraine—both on the territory of the country at war and for refugees in Poland is posted.

### Prayer Support, Spiritual Care and Pastoral Ministry

Prayer for peace in Ukraine began in hundreds of Catholic parishes and in dozens of monasteries of religious congregations in Poland already before the outbreak of war in Ukraine. Since the invasion of Ukraine by the troops of the Russian Federation, the prayer assault for peace in Ukraine and peace in the world flowed from almost all Polish Catholic churches, chapels, monasteries, and from thousands of Catholic homes in Poland. The first weeks of the war in Ukraine were full of a multitude of prayer initiatives for peace. Quantifying even a small portion of these initiatives is impossible. However, it should be noted in general terms that the clergy, consecrated persons, and lay faithful of the Catholic Church prayed (and continue to pray) for peace in Ukraine during Masses, devotions, rosary prayers, the Chaplet of Divine Mercy, the supplication “Holy God” and adoration of the Blessed Sacrament. In response to Pope Francis' appeal, thousands of Catholics in Poland undertook prayer and fasting for peace in Ukraine on Ash Wednesday (March 2). On Friday, March 25, 2022, a 24-h continuous adoration of the Blessed Sacrament—the so-called Adoration without borders—began in many churches in Poland. Many associations, such as the Light-Life Movement organized 24-h prayer for peace in Ukraine, youth ministries held all-night vigils under the slogan “Lord, save the brethren,” Rosary Jerichos, “Lenten prayer crusade.” In turn, the Sisters of the Congregation of Our Lady of Mercy at the Divine Mercy Shrine in Kraków-Lagiewniki invited people to cry out for God's mercy for all mankind and the restoration of peace in Ukraine by praying the Chaplet of Divine Mercy 24 h a day. On the initiative of the Polish section of the Pontifical Association to Aid the Church in Need, a special international rosary prayer campaign called “A Million Children Pray the Rosary” was organized for peace in Ukraine. On October 18, 2022, at the National Shrine of Our Lady of Fatima in Krzeptówki, the finale of the 17th edition of this prayer action “A Million Children Pray the Rosary” was held, in which 868,677 children around the world, including more than 230,000 children from Poland (Aid to the Church in Need, 2022, October 31) joined. A grassroots initiative was the action of spiritual adoption of Ukrainian soldiers—to pray for their intentions during the day.

A number of monasteries and parish churches in Poland have allowed Ukrainian refugees to receive the sacrament of confession in Ukrainian, and have begun celebrating Mass and services in the Eastern Rite and in Ukrainian, as well as Latin Rite Masses in Ukrainian (according to a 2018 Razumkov Center survey: 67.3% of Ukraine's population declared affiliation with one of the Orthodox Churches, 9.4% with the Byzantine Rite Catholic Church, 1.7% with the Latin Rite Catholic Church, 1.05% with Islam, while 11.0% declared themselves non-religious or unaffiliated (Ocoбливocтi peлiгiйнoгo, 2018). Sisters from cloistered orders in Poland undertook prayers for peace in Ukraine not only during the day, but also throughout the night.

On Friday, March 25, 2022, the Feast of the Annunciation of the Lord, during a penitential celebration in St. Peter's Basilica at the Vatican, Pope Francis consecrated Russia and Ukraine to the Immaculate Heart of Mary. At the same time, the same act—in conjunction with the Pope—was performed by a number of bishops in Poland.

Services and masses for peace in Ukraine and for Ukrainian refugees and volunteers were also celebrated by bishops and priests at tents of hope at border crossings. Most of the pilgrims who came to the national shrine of Our Lady of Czestochowa at Jasna Gora prayed for peace in the world. This intention also guided many other pilgrimages organized to regional and local shrines in Poland, of which there are now more than 800 (Mróz, 2019). Peace in Ukraine was also prayed for by Polish Caminos making pilgrimages on the Camino de Santiago trail network in Poland (more than 7,500 km of marked trail in Poland; Mróz, 2020) and outside Poland—in Spain, Portugal, France, Estonia, Latvia, and Lithuania.

An expression of the connectivity and solidarity of the Divine Mercy Shrine in Kraków-Łagiewniki with the Divine Mercy Shrine in Vinnytsia in Ukraine and the victims of the war in Ukraine was the bell of hope donated to the Vinnytsia Shrine. The bell is a gift of the shrine in Lagiewniki, and the ceremonial handover took place on October 2, 2022. It bears the words from St. John Paul II's homily delivered on August 17, 2002 at the Divine Mercy Shrine in Kraków-Łagiewniki: “Where war brings pain and death to the innocent, the grace of mercy is needed to soothe human minds and hearts and give birth to peace.” It should be added that since the war, bestial Russian troops have shelled the 370,000-strong city of Vinnytsia six times, killing 23 people, including three children (Divine Mercy Shrine in Kraków-Lagiewniki, 2022, October 5).

Hundreds of prayer initiatives have been published on social media as well. Parishes, monasteries, pastoral ministries, priests, monks and nuns, religious associations added overlays in the colors of the Ukrainian flag to their profile pictures on social media and published expressions of support and solidarity with Ukraine. Pastoral materials, texts of prayers and services in Ukrainian were developed and printed for Ukrainian refugees.

### Educational Assistance and Support to Improve Professional Skills

The clergy, religious, Diocesan and School Caritas Circles, volunteers, and faithful of the Catholic Church were also immensely involved in educational assistance to refugees from Ukraine. A major undertaking in this regard was the organization of free language courses, allowing refugees to learn the Polish language and to better integrate into their new country. Many priests, monks, and nuns who speak Ukrainian helped in the deployment of refugees, working with diocesan branches of Caritas.

According to estimates by the Ministry of Education and Science, in the final months of the 2021/2022 school year, there were 800,000 Ukrainian children in Poland, and about 200,000 Ukrainian children were studying within the Polish education system, including about 40,000 in kindergartens (about 500,000 to 600,000 children were studying remotely and connecting online with schools in Ukraine) (Ministerstwo Edukacji i Nauki, 2022). The new school year of 2022/2023, on the other hand, began with 185 thousand Ukrainian children in Polish schools. According to the Council of Catholic Schools, there are 493 Catholic schools in Poland (Rada Szkół Katolickich w Polsce, 2022). Religious sisters in Poland run 370 kindergartens, 100 elementary, secondary, and special schools, as well as 50 boarding schools, dormitories, and dormitories (Konferencja Wyższych Przełożonych Żeńskich Zgromadzeń Zakonnych, 2022a), while male orders, in turn, run 17 kindergartens and 121 schools. It has not been possible to obtain accurate data on the number of child refugees from Ukraine in Catholic elementary and secondary schools. However, it should be assumed that in most Catholic schools in Poland, care, including educational and recreational activities, cultural and musical activities and occupational therapy for children from Ukraine, have been organized.

In many Catholic schools, within the framework of the Caritas campaign called “BackpackFull of Smiles,” a collection of school supplies, clothing, sports equipment, as well as books (campaign under the slogan “Donate a book to Ukrainian children in Poland”) was organized for Ukrainian children in Poland and for children in Ukraine. Diocesan branches of Caritas in Poland organized in the Centers for Assistance to Migrants and Refugees (and continue to organize) adaptation and integration periods, educational and therapeutic workshops and classes, art classes and games and physical activities for children and adolescents—refugees from Ukraine.

In total, 14,800 children, including 4,000 refugee children from Ukraine, took part in Caritas' summer campaign. Currently, as part of long-term assistance in many Caritas day-care centers, excursions and educational integration activities are organized for children from Ukraine on days off from school, so that young refugees can learn about Polish culture, monuments and nature, practice the Polish language and integrate with Polish children."The first refugees arrived at our center already on the first day of the Russian aggression against Ukraine, that is, on February 24 around 11 p.m. Since our Caritas center is located close to the Polish-Ukrainian border, so at first those refugees who knew we were here – they already knew us before the outbreak of war - came to us. In the first weeks it was very difficult to coordinate this aid. This is because we received tons of materials and food from Poland and abroad. Everything had to be segregated, as the parcels included foodstuffs, as well as clothing, underwear, cleaning supplies and cosmetics. However, it was necessary to provide concrete – dedicated assistance to both refugees and, above all, material aid transferred to Ukraine in humanitarian transports. In the following weeks, refugees from the battlefields reached us, as well as the wards and caretakers of the center for the intellectually disabled. Currently, our support is more systemic. During the summer vacations, we organized two vacation camps for Ukrainian children, both from Ukrainian territories and refugee children staying in Poland. Caritas of the Rzeszow Diocese organized an integration point for refugees. Within the framework of this point, every other Saturday Ukrainian families come to us for whom we organize activities, games and excursions. Thanks to these activities, they get to know Poland, practice the Polish language and discover Polish traditions. Currently we are also conducting language, integration classes for children and young people from Ukraine". Agata Chmura—educator of the Caritas Recreation and Rehabilitation Center in Myczkowce (excerpted during an interview conducted on November 13, 2022).

Diocesan Caritas branches have also organized a number of workshops for Ukrainian women with children—including the Lublin Caritas branch's project “Supporting Moms. Improving professional and social skills of Ukrainian women.” The classes were conducted by specialists—career counselors, soft skills trainers, and psychologists, and aimed at raising professional and social competencies.

Catholic universities—the John Paul II Catholic University of Lublin, the John Paul II Pontifical University of Kraków, the Ignatianum Academy in Kraków and the Pontifical Faculty of Theology in Wroclaw—were also very actively involved in helping Ukrainian citizens who experienced the consequences of the Russian invasion. The Catholic universities provided material assistance (housing in student dormitories and financial assistance in the form of an allowance), legal assistance, spiritual assistance (services in Ukrainian and meetings with a pastor), psychological assistance, and childcare. In addition to this, students from Ukraine who met the relevant formal conditions, such as having Polish citizenship or a Pole card, were admitted to the university after qualifying by the relevant university committees. The authorities of the University and university staff in helping the Ukrainians were supported by students affiliated with the Student Government, scientific circles by organizing collections of foodstuffs, blankets, thermoses, quilts, pillows, thermal mugs, and warm clothing.

### Medical and Care Assistance in the Healthcare System

Work in the health care system and health pastoralism is one of the main activities of religious congregations in Poland. Health pastoral work is also undertaken to some extent by diocesan priests. Religious sisters, nuns, Catholic priests, and volunteers minister in hospitals, health centers and clinics, hospices, care facilities, nursing homes and community and parish nursing (Mróz & Roszak, 2022).

Religious orders in Poland whose charism is to help the sick (the Brothers of St. John of God, the Sisters Canonesses of the Holy Spirit de Saxia, the Elizabethan Sisters, the Orionists), as well as the Union of Polish Knights of Malta and the Maltese Medical Service, Caritas Poland and the Knights of Columbus joined in providing medical assistance to the Ukrainian refugees. The result of this assistance has been the provision of medical care to sick refugees from Ukraine, as well as the purchase and delivery of medical equipment to Ukraine: dozens of ambulances with equipment, specialized medical and surgical equipment, medicines, dressings, disinfectants, masks, drips, IV fluids, wound care products, and body coolers. Caritas of the Diocese of Zamość-Lubaczów is participating in the international project “Medicines for Ukraine,” the aim of which is to purchase and supply medicines for patients hospitalized in Ukrainian hospitals—purchase from the European Pharmaceutical Association with the assurance of compliance with the appropriate conditions, maintaining quality, effectiveness, and safety.

Refugees from Ukraine who are sick and have disabilities have also received free medical assistance from the Catholic Church in Poland in the healthcare system. This assistance was concentrated in the first days at medical points near border crossings and in hospitals operating in provinces close to the Polish–Ukrainian border. Hotlines began operating at Catholic medical facilities where refugees could obtain information in Ukrainian.

At the appeal of the Order of Malta, paramedics from the Maltese national unions of Germany, the Czech Republic, Belgium, England, France, Italy, Estonia, Lithuania, Spain, Austria, and Ireland came to Poland, bringing gifts for the refugees and performing several days of duty at points near the Polish–Ukrainian border and at the railway station in Kraków. At the appeal of the Order of Malta, paramedics from the Maltese national unions from Germany, the Czech Republic, Belgium, England, France, Italy, Estonia, Lithuania, Spain, Austria, Ireland, and observers from Malteser International came to Poland to bring donations for refugees and perform several days of duty at points near the Polish–Ukrainian border and at the PKP railway station in Kraków (Związek Polskich Kawalerów Maltańskich, 2022).

### Psychological, Legal, and Information Assistance

Images of homes being bombed, of innocent people being killed by Russian soldiers, have left deep marks on the psyche of many refugees. Hundreds of women and children who managed to escape from the war zones witnessed very tragic scenes, such as the death of their loved ones. When they managed to get to Poland, they felt safe, but in turn there was a concern for the future and constant anxiety caused by separation from their loved ones—fathers, husbands, and sons. In response to the traumatic experience and problems with adaptation to the new environment, many war refugees received specialized psychological assistance. This assistance was provided by many specialists—religious psychologists, priests, and volunteers in parishes, Caritas Poland, the Maltese Service, and the Knights of Columbus. Thanks to Caritas Poland alone, more than 24,000 refugees in Poland have benefited from information and legal assistance, and more than 1,000 from psychological assistance. Currently, many church institutions are preparing programs to coordinate and finance psychological therapy and stress and anxiety therapy for Ukrainian children, women, and their relatives.

The response of religious orders to the new needs of helping refugees are the integration clubs for Ukrainian refugees, which were established in Kraków and Harmęże thanks to the “Brother Sun” Foundation established in 2011 by the Province of St. Anthony and Blessed James Strzemię of the Order of Friars Minor Conventual (Franciscans) in Kraków. In the integration clubs, refugees can use the services of integration counselors who will help with administrative matters that, due to the language barrier or cultural differences, cause difficulties for people from Ukraine. Workshops are also held at these facilities to help refugees acquire basic professional qualifications and to enable them to find work in Poland.

Ukrainian refugees who arrived in Poland in the first weeks of the war have also been supported with legal and informational assistance. Caritas volunteers and diocesan branches helped the refugees with the translation of documents by certified translators, which was necessary for documents related to medical treatment, but also to confirm competencies obtained in Ukraine.

### Cultural and Sports Events to Support Refugees from Ukraine

It is also necessary to mention the many cultural, sports, religious, and tourist events aimed at helping refugees from Ukraine and assisting residents in Ukraine. On Divine Mercy Sunday, April 24, 2022, Poland organized the so-called Day of Good—the patronal feast of Caritas Poland—more than 700 events—charitable picnics, concerts, and festivals were held throughout Poland to collect donations and funds to help refugees from Ukraine and residents of Ukraine. During the 8 months of the war, many church institutions organized or co-organized a great many events in support of Ukrainians.

## Conclusions

The invasion of Ukraine by Russian Federation troops has caused the largest humanitarian crisis in Europe since World War II. Since the beginning of the war in Ukraine, i.e., from February 24 to October 31, 2022, according to the UN Office of the High Commissioner for Human Rights, there have been 16,295 civilian casualties, with 9,865 wounded and 6,430 (including 2,511 men, 1,716 women, 167 girls and 201 boys, as well as 34 children and 1,801 adults whose gender is still unknown) (The Office of the United Nations High Commissioner for Human Rights, 2022, October 31). The consequences of the tragic war in Ukraine—social, economic, political, and religious effects—are no longer only felt in Europe.

The article, on the basis of face-to-face interviews, observations, and analysis of statistical data, tries to reflect the geography of the Catholic Church's assistance in Poland in the first 8 months. This assistance covered as many areas of life as the ongoing war in Ukraine is destroying every day. The study's attempt to depict the first 8 months of this assistance confirmed that Church institutions, clergy, consecrated persons and the faithful of the Catholic Church in Poland have fulfilled their obligation to help their neighbors—helping refugees from Ukraine with the highest marks—fulfilling the Christian commandment “You shall love your neighbor as yourself” (Matthew 22: 39). It was crucial to help in the first month of the war, when more than 2 million refugees from Ukraine arrived in Poland. In the following weeks of the war, this aid continued, albeit with less intensity.

Now—in the eighth month of the Russian–Ukrainian war, refugees from Ukraine who have found a stable place to live in Poland are in need of help with their children’s education, support in the case of single mothers, and medical and psychological assistance. Professional psychological and therapeutic care for war refugees from Ukraine is urgent (Bouchard et al.,^2022^). It is also important to take care of refugees’ social networks and their support in religious life through chaplains and pastoral assistance and religious services in accordance with their religion (Oviedo et al., 2022). Religious congregations, clergy, Caritas Poland and numerous Church institutions of the Catholic Church are meeting these challenges and responding with concrete assistance. In the eighth month of the war, diocesan branches of Caritas, parishes, religious congregations, and Church institutions of the Catholic Church are initiating new projects to help refugees—organizing specialized centers to provide security for refugees, help with children's education, spiritual, psychological, and therapeutic support, assistance in deepening professional skills and finding work, and social integration of refugees with Poles in educational, sports, recreational, tourist, and entertainment activities. This assistance is primarily aimed at children with disabilities, mothers with young children, and the elderly. It is worth noting that Poles with disabilities are also involved in helping refugees with disabilities which has an additional therapeutic value for them (Santamaría Egurrola, 2022). In summary, the Catholic Church’s current assistance to war refugees from Ukraine, as well as to those fighting for the freedom of the Ukrainian country, is a strictly planned long-term assistance, the forms of which will probably change depending on the situation in Ukraine.

A separate and very important topic for further study is the research among priests, sisters, and religious brothers working in Ukraine, and who, after the outbreak of the war, overwhelmingly remained in Ukraine (there are about 700 priests from Poland (including 170 religious priests), 21 religious brothers and 332 sisters from Polish religious congregations working in Ukraine (Katolicka Agencja Informacyjna, 2022, April 13). It is they who support the residents, provide spiritual, material and psychological care, evangelize, bring hope and peace to their hearts. It is necessary, therefore, that in the study of geography and religion and health, not to overlook such an important issue as care—the geography of care, a sense of brotherhood—the geography of brotherhood, and ideas of loving one’s neighbor in these difficult modern times.

## Study Limitations

It should be emphasized that the analyzed research on the Catholic Church's assistance to refugees from Ukraine in Poland does not represent a representative size in the quantitative sense, remaining limited to official data presented by selected units of the Catholic Church in Poland. The key is to capture them qualitatively, made possible by our own research—in-depth interviews and observations. It is impossible to determine in detail the volume of aid expressed in monetary amounts and the number of beneficiaries—although the work presented here refers repeatedly to quantitative data from institutional sources. It would certainly be interesting to compare these results with the assistance of the Churches—not only the Latin Church—in countries neighboring Ukraine to which refugees have poured into (e.g., Romania, Moldova, Slovakia, and Hungary).
